# Experiencing beauty in everyday life

**DOI:** 10.1038/s41598-024-60091-w

**Published:** 2024-04-24

**Authors:** Anna Lena Knoll, Tristan Barrière, Rosalie Weigand, Thomas Jacobsen, Helmut Leder, Eva Specker

**Affiliations:** 1 Department of Cognition, Emotion, and Methods in Psychology, University of Vienna, Vienna, Austria; 2 Experimental Psychology Unit, Helmut Schmidt University/University of the Federal Armed Forces Hamburg, Hamburg, Germany; 3 Vienna Cognitive Science Hub, University of Vienna, Vienna, Austria

**Keywords:** Human behaviour, Psychology and behaviour, Cultural evolution, Coevolution

## Abstract

Beauty surrounds us in many ways every day. In three experience sampling (ESM) studies we investigated frequency, category of eliciting stimuli (natural vs human-made) and, the potential moderating role of several individual difference measures on such everyday experiences of beauty in an ecologically valid manner. Further, we explored the impact of such experiences on valence & arousal. Study 1 re-analysed data from a previous study, in line with the current aims. In Studies 2 and 3, we asked participants to report daily experiences of beauty using a mixed random and event-contingent sampling schedule. Mobile notifications (random sampling) prompted participants to take a photo and rate the beauty of their surroundings. Further, current valence and arousal were assessed. Notification frequency and total days of participation differed between these two studies. Participants were able to report additional experiences outside of the notification windows (event-contingent sampling). Our results indicate that we frequently encounter beauty in everyday life and that we find it in nature, in particular. Our results further suggest a mood-boosting effect of encounters with beauty. Lastly, our results indicate influences of individual differences however, these were inconclusive and require further attention.

## Introduction

Beauty surrounds us; we may seek it out intentionally or happen upon it by accident. Humans’ ability to perceive and appreciate beauty, their sense of beauty, has been found to have a number of influences, including guiding attention^1,2^, eliciting and enhancing emotions^3,4^, and reducing stress^5^. But why are we able to see and appreciate beauty in the first place? And what determines what we find beautiful?

Evaluations of beauty are at least partly subjective^1^, but there appears to be stronger agreement for natural (e.g., faces) than human-made stimuli (e.g., art)^1,6^. From an evolutionary standpoint, our ability to notice and appreciate beauty must have served a purpose in our evolutionary past or present. Natural stimuli—such as faces—are evolutionarily relevant in that, for instance, their symmetry might be an indicator of genetic quality^7^. Yet, symmetric faces are also perceived as more beautiful. Consequently, choosing a mate with a “beautiful face” has often been argued to also help us choose the mate with better genes.

For human-made stimuli (e.g., art), evolutionary functions are less obvious. But does that mean that beauty seen in human-made objects is simply a by-product? Verpooten^8^ describes two opposing evolutionary accounts: (1) The standard evolutionary account of aesthetics that argues for direct selection, i.e. beauty helps us in selecting suitable mates (as in the example above) and environments. Beauty in human-made objects is simply a by-product. (2) An opposing co-evolutionary account which proposes indirect selection by which aesthetic preferences shape aesthetic traits, such as the peacock’s tail. Beauty (or having a large tail) may start as an “honest cue” (larger tails = stronger mate) but then becomes a goal in and of itself (tail length is no longer indicative of mate quality). Beauty in human-made objects is a result of this runaway process^8,9^.

Currently, the literature mainly supports the standard account. This account suggests that people see beauty mostly in nature. Accordingly, a number of studies have shown preferences for natural landscapes over human-made environments^6,10,11^, or what has been termed *biophilia*^12^: a tendency to prefer life or lifelike objects and environments. Viewing such stimuli can affect emotions and well-being positively (e.g., increase happiness^13^).

Most people, however, spend more time in human-made environments, with urban areas estimated to house 60% of the world population by 2030^14^. While urban areas are often limited in their access to nature they still hold great potential for beauty. Within urban environments some elements are more likely to be seen as beautiful than others. For example, a study in Malaga, Spain^15^ found leisure sites, sites of cultural history, and places with panoramic views to be more appreciated than residential and industrial areas. Similar sites can be found in many cities, though their specific appearance may vary from city to city. Further, depending on the time of day, skylines may be appreciated as much as nature. Specifically, night-time skylines and natural landscapes are rated higher than daytime skylines^16^. Yet, human-made environments with natural features (e.g., trees) are often preferred over those without^17–19^.

Nonetheless, these findings cannot differentiate if human-made beauty is a by-product (standard account) or an end-result (co-evolutionary account). Furthermore, the role of individual differences remains unclear.

Verpooten^8^ aims to unite these two opposing views by focusing on cultural evolution through social learning. He suggests that acquired art knowledge will moderate aesthetic preferences. Consequently, more art knowledge would result in stronger preferences for human-made aesthetic objects (supporting the co-evolutionary account). For people who have not acquired this knowledge, preferences are based on their evolutionary usefulness (i.e., standard account).

Notably, Verpooten’s^8^ suggestion focuses specifically on understanding aesthetic appreciation of (visual) artworks. However, there are many other human-made stimuli in everyday life that we can appreciate for their aesthetic value. Following the idea of cultural evolution, knowledge besides that of art may be relevant to the appreciation of everyday (human-made) aesthetics. For instance, forming connections to city environments (city relatedness) by experiencing urban life may shape aesthetic preferences towards the human-made direction, and connections to nature (nature relatedness) may strengthen the natural tendency for biophilia.

*Nature relatedness* (NR) entails people’s appreciation (including aesthetic), knowledge, and experience of nature^20–22^. Since aesthetic appreciation (of nature) is part of NR it may affect what people find beautiful in their everyday lives. While exposure to nature can increase NR^23^, many people are mainly exposed to urban environments. Thus, instead of, or in addition to, NR, people may develop *City Relatedness* (CR), referring to attitudes towards and experiences of city environments. Both can influence how we perceive the world around us^17^ and thus may affect evaluations of beauty in everyday (urban) environments.

Finally, these accounts are domain-specific, however, our ability to perceive beauty can also be seen as domain-general. Here, appreciation of beauty is conceptualised as trait(-like) emotional and cognitive involvement with beauty^24,25^, on its own as *Engagement with Beauty*, or as part of the Big-5 personality trait ’Openness to Experience’^24,26^. While everyone can appreciate beauty, someone with a high level of engagement may find more beauty within everyday life by intentionally seeking it out or simply noticing it more. People who have a higher appreciation for beauty in one domain (e.g., art) generally also have it in other domains (e.g., music)^26,27^. However, it is also possible for people to have more appreciation for one domain than another^27^.

Limiting the research discussed thus far is that it has mainly been conducted in controlled laboratory settings. However, to make evolutionary sense a crucial assumption of all evolutionary perspectives is necessary: Our ability to perceive beauty is adaptive only with relevance to our daily lives. While everyday stimuli can and have been used in lab settings this fails to capture the impact and embeddedness of encounters with aesthetics, especially beauty, within our daily lives. It is unclear how common aesthetic experiences are (in everyday life) or what their quality is. Likely, when we think of “aesthetic experience” in such a context, we should not think in terms of extremely moving artworks, but rather of walking down the street and noticing a tree in bloom.

Therefore, we aimed to investigate the frequency and quality of aesthetic experiences in daily life. Additionally, based on the theoretical concerns discussed above, we investigated how such experiences are influenced by category (i.e., human-made vs natural) and individual differences (art expertise, city and nature relatedness, engagement with beauty, and personality).

We hypothesised that: (1) aesthetic experiences, of beauty in particular, are frequent in everyday life, (2) natural stimuli are more likely to elicit aesthetic experiences than human-made stimuli, (3) this will be moderated by individual differences. Lastly, as it has previously been shown that viewing art^5,28,29^ and nature^13^ can affect well-being, we explored influences of experiencing beauty on affective states (4) .

We started by re-analysing a previous study^30,31^ (Study 1) and then collected additional data (Studies 2 and 3). All studies used an experience sampling (ESM) approach, allowing the documentation of daily aesthetic experiences in an ecologically valid way^32^. While this (unlike a lab setting) allows for no control over what participants are experiencing, it enables capturing naturally occurring experiences^33^. It thus aligns with our aims, as this approach enables capturing the frequency and quality of daily aesthetic experiences. In our case, each participant responded to multiple ESM questionnaires per day across multiple days, allowing for collection of substantial amounts of data in a relatively short period and letting us investigate how such experiences differ within and between people.

## Study 1

The data re-analysed in Study 1 came from a study investigating the influence of dispositional affect on aesthetic experiences (frequency, intensity, and eliciting stimulus) in two ESM studies. As such, this data was suited to address the frequency of aesthetic experiences (H1) and the eliciting stimulus (H2). The main finding from these two previous studies is that higher dispositional affect is related to higher intensity but not frequency of aesthetic experiences. Thus these findings also inform H3, suggesting at least some influence of individual differences on daily aesthetic experiences, although this seems only a qualitative and not a frequency influence.

### Methods Weigand & Jacobsen 2022

Random notifications occurred either 12 (Study 1a) or 4 (Study 1b) times per day for 14 days. At each measurement time point, participants reported whether they had an aesthetic experience within the past hour. They were given the following definition of aesthetic experience: ‘An aesthetic experience is the reception and evaluation of an object or sensorial entity with respect to one or more relevant concepts (such as beauty, elegance, rhythm, and so forth)’^30^. Next, participants specified the elicitor of the experience by picking from 8 predefined categories (i.e., visual art, performing art, music, literature, nature, humans, inanimate object, other). The intensity of the reported experiences was measured in terms of the savouring (Study 1a, ‘I savoured the present moment’, ‘I was thinking about things that make me feel happy’, ‘I was thinking about things that make me feel pleasure’) or in terms of aesthetic emotions (Study 1b) using 10 items of the pleasing, prototypical aesthetic, and epistemic aesthetic emotions subscales of the Aesthetic Emotions Scale (AESTHEMOS)^34^. Additionally, a 6-item short scale^35^ was used to measure mood (valence, calmness, and energy) in Study 1b, irrespective of whether an aesthetic experience was reported at the given measurement time. Dispositional affect was measured using the Positive and Negative Affect Schedule (PANAS)^36^ and the Dispositional Positive Emotions Scales (DPES)^37^.

The participant sample consisted of 99 people (women = 47, men = 52) in Study 1a and 97 (women = 40, men = 57) in Study 1b; All, except 10 participants in Study 1b, were students at Helmut Schmidt University/University of the Federal Armed Forces Hamburg (see Weigand and Jacobsen, 2022^30^ for details).

### Re-analysis

The predefined categories (excluding the “other” category) used to specify the content of aesthetic experiences can be sorted into the broader categories of human-made (i.e., visual and performing art, music, literature, inanimate object) and natural (i.e., nature and humans). This allowed for a re-analysis of the existing data to investigate our current research questions.

The re-analysis was done in IBM Statistics SPSS for Mac (vs 27, IBM Corp., Armonk, NY, USA). Due to the hierarchical structure of the data (ESM responses at level 1 nested within subjects at level 2) we used multilevel modelling MLM to re-analyse the data. In contrast to ordinary least squares (OLS) regression, MLM uses multiple error terms to partition the variance between the different levels of data which allows analysing within and between level relationships without violating standard independence assumptions^38^. The multilevel models are fitted to the data using maximum likelihood estimation. This approach can handle missing and unbalanced data. For the analyses, the level-1 variables were group-mean centred; level-2 variables were grand-mean centred. All analyses employed the conventional .05 alpha level.

We constructed multilevel models to investigate the effect of category (i.e., natural vs human-made) on (1) the intensity of aesthetic experiences in terms of savouring, (2) aesthetic emotions, and (3) state affect.

### Results

#### Frequency

The original study^31^ reported a high frequency of aesthetic experiences: participants reported aesthetic experiences in the previous hour 35% of the time (SD= 20%, range = 3-94%) over the course of two weeks, or roughly four times a day (range = roughly one experience per 3 days to almost 12 experiences per day). The most common reported categories were nature (22%) and human beings (20%)—both falling within the broader category of natural; human-made categories occurred less frequently (performing arts—16%, inanimate objects—15%, music—13%, literature—5%, and visual arts—2%); 6% of responses were categorised as “other”. Grouping categories into our broader categories (excluding the “other” category), we find 42% natural and 51% human-made experiences.

#### Savouring of aesthetic experiences

An effect of category on the intensity of aesthetic experiences was observed: higher levels of savouring were reported for the natural (vs. human-made) category domains (b $=$ 0.33, p < 0.001).

#### Aesthetic emotions

Higher levels of aesthetic emotions were reported in response to natural stimuli (b $=$ 0.08, p $=$ 0.010). Specifically, participants reported higher levels of prototypical aesthetic emotions (e.g., awe, wonder^34^) in response to natural domains (b $=$ 0.13, p < 0.001). In contrast, natural stimuli resulted in lower levels of epistemic aesthetic emotions (b $= -0.13$, p $= 0.002$)—emotions related to making sense or meaning of the experience (e.g., interest, surprise)^34^.

#### State affect

Experiences categorised as natural were associated with higher state valence (b $= 0.63$, p $< 0.001$), energy (b $= 0.62$, p $<0.001$), and calmness (b $= 0.51$, p $< 0.001$).

## Discussion Study 1

These results provide first evidence for H1 and H2: Aesthetic experiences seem common in daily life, with nature being a frequent elicitor. Although experiences were overall more commonly human-made (51%) than natural (42%), the most common subcategories within these broad categories were natural. Additionally, experiences associated with nature were more intense. Building on this, we collected data for Studies 2 and 3 that aimed to specifically test H1 and H2 as well as H3 (looking at individual differences).

In Study 1, participants were asked to determine whether they “had an aesthetic experience” within the past hour. This likely requires the experience to be deep enough to register consciously. This may not always be the case (see tree in bloom example): Along a spectrum of depth, length, and breadth, many of our everyday experiences are likely brief and shallow, however they are still present (we are perceiving the world around us at any given point in time and respond to it in one way or another)^39^. In Studies 2 and 3 we set out to capture such more shallow experiences as they occur. A crucial difference was thus that we asked participants to take a picture of their environment and then rate its beauty. This brings potentially shallow, unconscious experiences into focus by changing their depth, thus making them measurable. To explore the frequency of these experiences in further detail, we used different numbers of sampling points in Studies 2 and 3, resulting in approximately 1 or 3 measurements per hour.

## Study 2

### Methods

#### Participants

We collected data from 106 participants in a pre-ESM lab session; 4 dropped out during the ESM part of the study; 1 was excluded due to submitting photos that were not taken in the moment (verified by EXIF data); and 16 participants were excluded due to low response rates to random ESM notifications ($<65$%). Three participants would have been excluded based on the 65% criterion but made up for it with event-contingent responses. Thus, data from 85 participants (70 women, 15 men) was available for data analysis. All participants were fluent in German, between the ages of 18–30 (mean = 21.3, SD = 2.52), and Psychology students recruited through the online system of the Faculty of Psychology of the University of Vienna. The study was carried out in accordance with the Declaration of Helsinki and was approved by the ethical committee of the University of Vienna. Participants gave written informed consent before participation and received class credit for their participation.

#### Individual difference measures

We collected data to measure individual differences in Art Expertise, Nature Relatedness, City Relatedness, Engagement with Beauty, and Personality. Art Expertise was assessed using the Vienna Art Interest and Art Knowledge questionnaire (VAIAK)^40^. NR was assessed using two scales: the 21-item Nature Relatedness Scale (NRS)^22^ with three subscales (self, experience, and perspective) and the Nature Connection Index (NCI)^21^. We used the City Relatedness Scale (CRS)^17^ to assess CR. This scale was constructed to mirror the items of the experience and self subscales of the NRS in a way that makes them apply to a city environment. Further, we used the Engagement with Beauty Scale (EBS)^24,25^ which includes three subscales assessing natural, artistic, and moral beauty. Lastly, a 10-item Big-5 personality questionnaire (BFI-10^41^) was included. All questionnaires were provided in German. The NCI was translated by the first author and checked by the second and last author as well as an independent native German speaker, although, this translation has not been formally validated. We were provided with a German translation of the NRS^42^. However we made some changes to this translation to better reflect the English original. Again, translations were done by the first author and then checked and discussed with the second and last author as well as an independent native German speaker.

#### Experience sampling

Participation in the experience sampling part of the study required that participants installed the *Samply Research*^43^ app on their personal smartphones to allow control of ESM notifications. We used both random and event-contingent sampling: Participants received notifications throughout the day (random sampling), each containing a link to a questionnaire measuring the occurrence of experiences of beauty. An additional link to the same questionnaire was permanently available within Samply and was instructed to be used to report experiences (event-contingent sampling) outside of the random notification windows. The questionnaire itself was implemented in Qualtrics (https://www.qualtrics.com/).

At each ESM measure, participants were first asked to take a photo and/or describe their current surroundings. Next, they were asked whether they were paying attention to their surroundings as a whole or a specific object within those surroundings. Subsequently, a categorisation of the surroundings or object into “mostly human-made” or “mostly natural” was made, followed by a beauty rating (7-point scale ranging from “not beautiful at all” to “very beautiful”) of the surroundings or object. Lastly, participants were asked how they were currently feeling. This was rated on two 7-point scales ranging from “negative” to “positive” (valence) and from “calm” (German: “ruhig”) to “excited” (German: “aufgeregt”) (arousal). Here we aimed to keep the ESM measure brief and thus opted for only one question (as opposed to the 6-item measure in Study 1b) for valence and arousal with commonly used German anchor points for each.

A short follow-up questionnaire was administered a day after participants completed the main ESM part. Here, participants were asked about where they spent their time during the study. Specifically, whether most of it was spent in Vienna or not and how much of their time they spent in mostly human-made as compared to mostly natural surroundings. This was done in order to have more information about the participants’ general environment which we assumed influences the frequency of reported experiences being classified as human-made or natural. Further, participants were asked whether the way they looked at their surroundings had changed since the start of the study and, if so, how.

**Figure 1:**
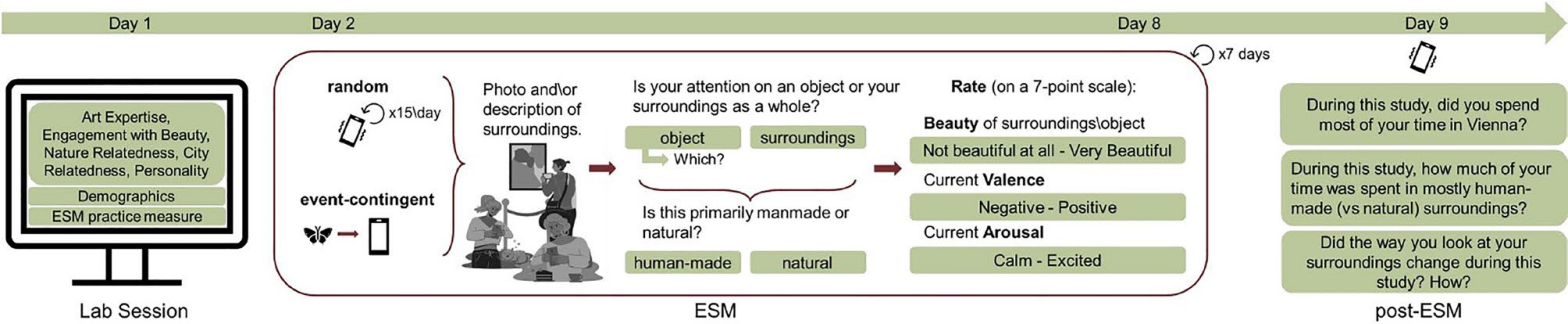
Procedure illustration: each participant started the study with a lab session in which we collected demographic data and individual difference measures. An ESM practice measure ends the lab session. The main ESM part started the next day. Participants received 15 random notifications daily for 7 days, each prompting them to respond to the same questionnaire pertaining to current experiences of beauty. A permanently available link allowed participants to report additional experiences (i.e., event-contingent sampling) outside of the random notification windows. The day after completing the ESM, participants received a notification for a follow-up questionnaire. (Illustrations are from Canva under their free content license).

#### Procedure

Each participant was invited to a lab session before the ESM investigation (see Fig. 1 for an illustration of the procedure). Here, we first provided participants with information about the study procedure. We then collected demographic information and the individual differences measures (see above) in the following order: VAIAK & demographics, EBS, NR-21, NCI, CR, BFI-10. Subsequently, participants received instructions on the ESM part of the study. They were asked to install Samply on their smartphones and then sign up for the ESM study by scanning a QR code and using a participant ID received during the lab session. Through Samply participants received notifications containing a link to the ESM questionnaire. The first notification was set to occur 2 minutes after sign-up, while participants were still in the lab. This measure was intended to familiarise participants with the usage of the app and the ESM procedure.

The main ESM part started the day after the lab session: for a week, participants were prompted to report experiences of beauty 15 times per day (i.e., random sampling). All ESM notifications occurred at random intervals between 9 am and 10.30 pm and had a 20-minute response window. We instructed participants to respond to as many notifications as possible and to use the event-contingent option to report additional experiences. For event-contingent sampling, we explicitly instructed participants to only fill out the survey once in the case of repeated aesthetic experiences with the same stimulus (e.g., continuously enjoying your houseplant).

The day after the main ESM part was completed, participants received a notification at 12 pm including a few follow-up questions. During the practice measure in the lab session and the post-ESM measure, participants were asked whether they wanted to receive a collage of their most beautiful (i.e., highest ratings) photos. If they indicated ’yes’ at either of those times, collages were prepared in R and participants received a personal download link through Samply.

#### Analysis

We analysed data in R (R v4.2.0, R Studio v2022.2.1.461)^44,45^, employing the LME4^46^ and lmerTest^47^ packages to perform multilevel analyses and the GGplot2 package^48^ for data visualisation.

We first constructed a multilevel model to investigate whether experiences of beauty are affected by category. This model included by-participant as the random intercept and category as fixed effect term. Category was dummy coded as 0 (= human-made) and 1 (= natural). To check the model fit we compared this “category model” to the “null model” (i.e., model with by-participant random term only).

Next, we addressed the influence of individual differences on experiences of beauty. We set up 3 multilevel models by extending the fixed effect part of the “category model” with the individual difference measures and possible interaction terms:

- Aesthetics model. Included VAIAK (interest and knowledge) and EBS scores.
- Nature and city relatedness model. Included NR, NCI, and CR scores.
- Personality model. Included the BFI scores of each Big-5 facet.

For all models the scores of the included individual difference measures were grand-mean centred. Model fit was checked by comparing each of the three models to the “category model”. Note that BFI scores for one participant were partly missing. Thus, the “personality model” was run with N $=$ 84 participants; to be able to calculate model fit the “category model” was also re-run with N $=$ 84.

Lastly, we were interested in potential effects of beauty on affective state. We thus, ran two multi-level models; one for each of our affect measures (i.e., valence and arousal). Both models included the beauty ratings as the fixed effect term and by-participant and by-day random intercepts. Beauty ratings were centred on the person-level mean before setting up the multilevel models. Model fit indices were calculated by comparing each model to its corresponding null-model.

For all models, the model fit indices are reported in the supplementary section.

### Results

#### Response behaviour

Participants responded to an average of 78.6% (SD = 7.81, see suppl. Table 1) ESM notifications, taking 1–2 min (mean = 78.7 s, SD $=$ 29.7) to complete the questionnaire. They typically responded within 4.07 min (SD $=$ 1.39) after receiving a notification. 35.29% of participants used the event-contingent response option at least once (mean $=$ 1.79, SD $=$ 5.09, max $=$ 38).

#### Frequency of experiences of beauty

**Figure 2:**
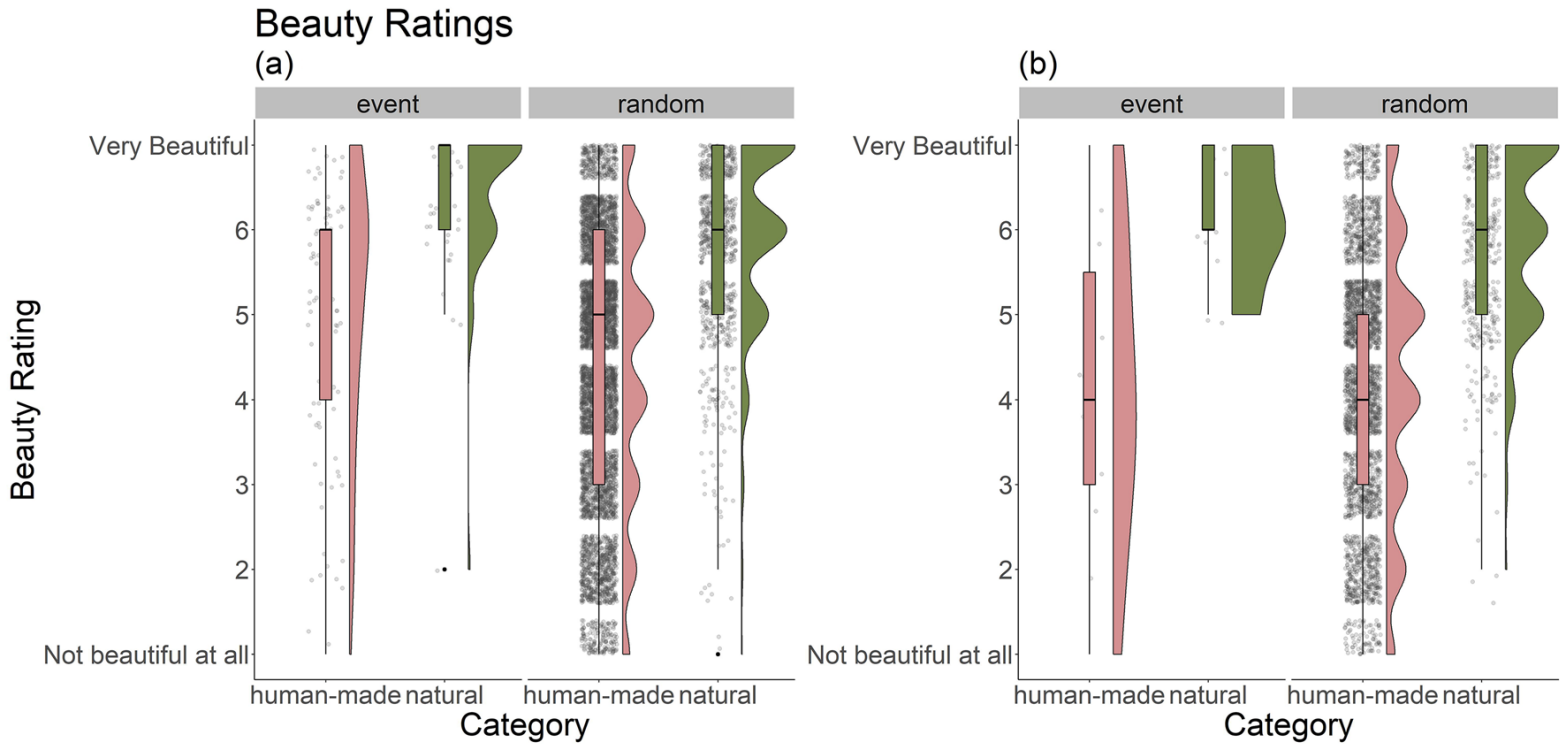
Beauty ratings in Study 2 (**a**) and 3 (**b**) grouped by Category (i.e., human-made vs. natural) and type of the response (i.e., event-contingent vs. random notification). Beauty ratings tended to be higher for natural than for human-made experiences. Event-contingent responses were overall rated higher in both Study 2 and 3. For Study 2 this was the case in either category. Jittered dots represent individual responses; here we can observe that (1) participants rarely used the event-contingent option, and (2) that human-made experiences were more common than natural ones.

Participants frequently gave high beauty ratings (mean = 4.63, Median = 5, SD = 1.68), most ranging between 4-6 (see Fig. 2). Specifically, 74.8% of 7163 total responses were rated 4 (N = 1213) or higher (N = 4142). In line with H1, this suggests that aesthetic experiences are indeed frequent in everyday life.

#### Influence of category

In our everyday environment we more often encounter human-made than natural objects and surroundings—participants classified 84.9% (SD = 9.20) of experiences as human-made. Correspondingly, participants estimated spending 85% (SD = 9.82) of their time in human-made environments in the post-ESM questionnaire.

A closer look at the beauty ratings shows that natural stimuli received higher ratings (mean = 6.01, median = 6, SD = 1.07) than human-made ones (mean = 4.38, median = 5, SD = 1.65). Further, event-contingent responses received higher beauty ratings (mean = 5.59, SD = 1.52) than random responses (mean = 4.61, SD = 1.68), both in the human-made and natural category (see Fig. 2a). As only 30 participants used the event-contingent option, response type was not considered in our multi-level models. However, we speculate that the event-contingent option was primarily used when something was consciously noticeable as beautiful and thus also likely to receive a high rating.

Supporting H2, results of the multilevel analysis confirm a significant relationship between experiences of beauty and category—natural experiences were rated more beautiful than human-made ones (b = 1.64, p < 0.001, suppl. Table 2).

#### Individual differences

To analyse the role of individual differences (see suppl. Table 6 for mean scores), we grouped our individual difference measures setting up three multilevel models—an aesthetics model, a nature and city relatedness model, and a personality model.

The aesthetics model showed a negative effect of art knowledge (b $= -0.08$, p = 0.035) and a positive effect of category (b = 1.63, p < 0.001). Thus, beauty ratings were lower in people with higher knowledge. As such, gaining art knowledge seems to create a more critical viewer, and thus lower ratings overall. The effect of category is the same as above, i.e., experiences within the natural category were rated higher than those in the human-made category.

**Figure 3:**
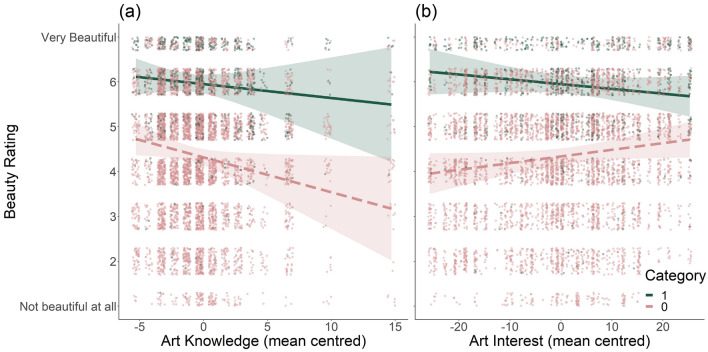
Significant two-way interactions found in the “Aesthetics Model” in Study 2. Specifically, category (0 = human-made, 1 = natural) interacts with art knowledge (**a**) in that ratings tend to be higher in both categories with lower levels of art knowledge. The interaction of category and art interest (**b**) shows an increase in beauty ratings with higher levels of art interest in the human-made category and a decrease in the natural category. Regression lines and 95% confidence intervals are shown; coloured dots represent individual observed beauty ratings.

To explore the role of individual differences (H3) we looked at the two-way interactions between category and knowledge, interest, and EBS; 3- and 4-way interactions (suppl. Table 3) will, for brevity, not be discussed. We found two relevant significant interactions: a positive interaction between category and art knowledge (b = 0.05, p = 0.023) and a negative interaction between category and art interest (b $= -0.03$, p < 0.001). Beauty ratings were generally higher in both categories with lower levels of art knowledge (see Fig. 3a). Nonetheless, the interaction indicates a less pronounced effect in the natural category than the human-made category, again potentially reflecting a more critical attitude that may be easier to apply to human-made stimuli. That said, the pattern of effects is the same across categories.

In contrast, art interest showed a differing pattern between categories (see Fig. 3b). Specifically, with higher levels of art interest beauty ratings increase within the human-made but decrease in the natural category. Potentially, an increased interest in a specific category of human-made objects (such as art) may make it easier to see beauty in human-made objects in general. The decrease for the natural category may reflect, similar to the effect of art knowledge described above, a slightly more critical stance—rather than considering all/most nature beautiful, judgements may be more nuanced.

The nature and city relatedness model showed only a significant effect of category (b = 1.61, p < 0.001, see suppl. Table 4).

The personality model showed a significant effect of category (b = 1.6, p < 0.001), a significant two-way interaction of category and Neuroticism (b $= -0.17$, p = 0.01), and several significant 3 (or more)-way interactions (suppl. Table 5). Visually the two-way interaction displays a similar, though less pronounced, pattern to the interaction between category and art interest (see suppl. Fig. 1)—with higher levels of Neuroticism, natural stimuli were rated (slightly) less and human-made ones as more beautiful. Contrary to what we expected, no two-way interaction with Openness to Experience was present. However, this is consistent with the results of the aesthetics model above. Both openness and EBS (as a more extensive measure of the aesthetic appreciation sub-facet of openness) only appear relevant in 3 (or more)-way interactions.

#### Influence of experiences of beauty on affective state

Lastly, we analysed the influence of beauty on valence and arousal.

**Figure 4:**
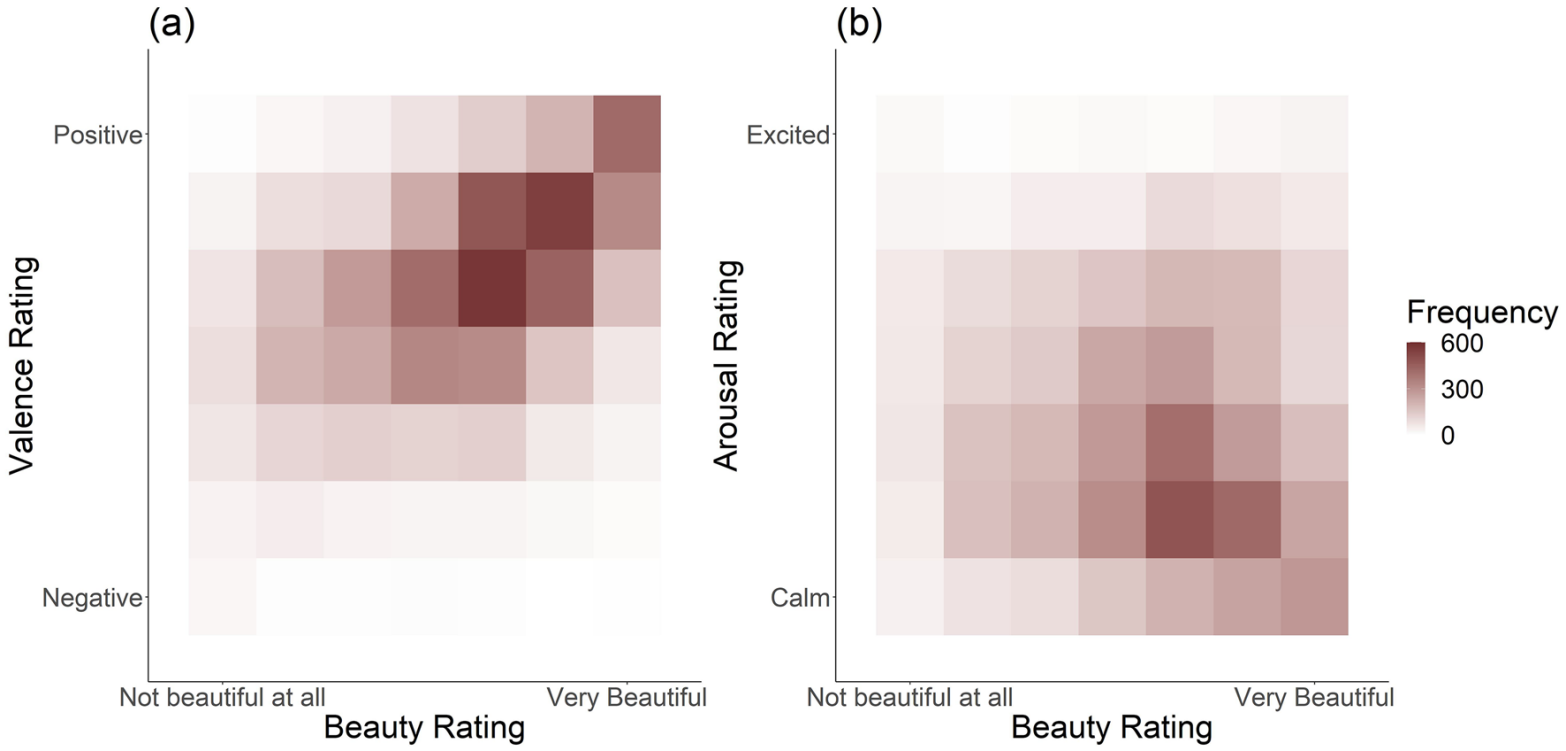
Study 2: shows frequency of occurrence of valence (**a**) or arousal ratings (**b**) in relation to different beauty ratings. Higher beauty ratings appear to more often go along with higher valence and lower arousal ratings. Overall, valence ratings tend to be medium to high; arousal ratings tend to be rather low or neutral.

The two multilevel models show that beauty impacts both valence and arousal (also see Fig. 4; suppl. Table 7). Specifically, more beautiful experiences lead to higher valence (b = 0.29, p < 0.001); arousal was negatively influenced by beauty (b $= -0.09$, p < 0.001)—people appear to feel calmer during more beautiful experiences.

## Study 3

Study 3 was a more intensive variation of Study 2, additionally focusing on repeated experiences. We increased the number of notifications per day, but reduced days of participation.

### Methods

#### Participants

We, again, recruited Psychology students through the online system of the Faculty of Psychology of the University of Vienna. Of 52 participants who took part in the lab session, 1 dropped out during the ESM part of the study, 5 were excluded due to low response rates (<65%), and 1 was excluded due to misunderstanding part of the instructions. This left a total of 45 participants (30 women, 14 men, 1 non-binary) for the analysis. All participants spoke fluent German and were between the ages of 18 and 37 (mean = 23.4, SD = 3.97).

#### Measures and procedure

Measures and procedure for the pre-ESM lab session and the follow-up questionnaire remained the same as in Study 2. Some major changes were made to the ESM part:

- The number of days was decreased from 7 to 2.
- The number of random notifications was increased from 15 to 50 per day.
- The response window for each notification was adjusted to 10 min to account for the higher notification frequency.
- The ESM questionnaire was extended by one question. Specifically, we asked participants whether the currently reported object/surrounding was the same as in the preceding ESM response. This was done because, due to the increased notification frequency, it was more likely that participants were still experiencing the same stimuli across sequential responses. This new question was added between the object/surroundings question and the categorisation (human-made/natural) question.

#### Analysis

We performed analyses analogous to those in Study 2. We ran three additional models by including repetition as a fixed effect term in the category, valence, and arousal models. To get model fit indices (results reported in the supplementary section) these were compared to corresponding models not including repetition.

### Results

#### Response behaviour

The mean ESM response rate was 80.4% (SD = 9.33), somewhat higher than in Study 2. Most people took just over a minute to complete each ESM questionnaire (mean = 71.7 s, SD = 25.0, suppl. Table 1) and typically responded within 2 min (SD = 1.9). Only 11.11% of participants used event-contingent responses (mean = 0.44, SD = 1.59, max = 9). This may be due to the increased sampling rate, as random sampling would be less likely to miss an event worth reporting.

#### Frequency of experiences of beauty

Beauty ratings were frequently high (mean = 4.43, median = 5, SD = 1.65); 72.18% of responses were rated 4 (N = 723 of 3634 total responses) or higher (N = 1900). While this is a slightly lower percentage than in Study 2, it still suggests that aesthetic experiences are frequent in everyday life.

As people often spend extended periods of time in one place, we also checked for repeatedly experienced stimuli. Indeed, participants reported 25.5% (SD = 15.7) of experiences as repeated.

#### Influence of category

**Figure 5:**
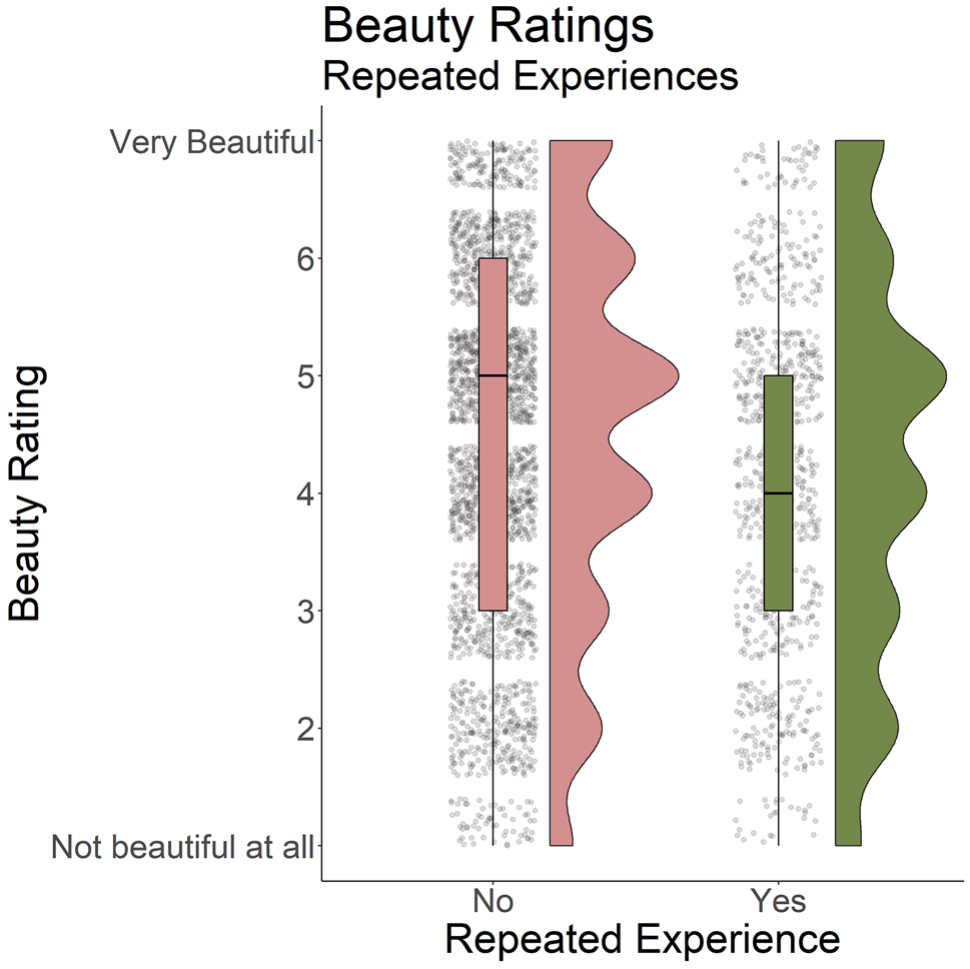
Study 3: beauty ratings grouped by whether they were repeated or not. Repeated experiences tended to receive lower beauty ratings than non-repeated ones. Jittered dots represent individual responses.

Beauty ratings resemble those in Study 2—while human-made stimuli were encountered more often (mean = 86.3%, SD = 9.37), they received lower ratings (mean = 4.2, median = 4, SD = 1.61) than natural ones (mean = 5.9, median = 6, SD = 1.06, see Fig. 2b). Event-contingent responses received higher beauty ratings (mean = 5, SD = 1.75) than random responses (mean = 4.43, SD = 1.65). Additionally, repeated experiences received lower ratings (mean = 4.23, SD = 1.68) than non-repeated ones (mean = 4.5, SD = 1.64, see Fig. 5).

Study 3 replicated the results of Study 2: Natural experiences received higher beauty ratings (b = 1.64, p < 0.001, suppl. Table 8) than human-made ones. An additional multilevel model showed that repeated experiences received lower beauty ratings (b $= -0.17$, p = 0.006, suppl. Table 9); this did not depend on whether the repeated experience was natural or human-made, as the interaction between category and repetition was not significant (b = 0.34, p = 0.086).

#### Individual differences

Result of all three individual difference models diverge from Study 2.

First, in the Aesthetics Model, none of the significant interactions found in Study 2 held. Instead we observed a significant interaction between art knowledge and interest (b $= -0.0086$, p $=$ 0.004); Category remains significant (b = 1.63, p < 0.001, suppl. Table 10).

Next, in contrast, to Study 2, the nature and city relatedness model shows significant interactions between the NR scales and the CR scale (NR*CR: b $= -1.37$, p = 0.03; NCI*CR: b = 0.026, p = 0.028). In addition we found a significant effect of category (b = 1.57, p < 0.001), as well as three- and four-way interactions (suppl. Table 11).

**Figure 6:**
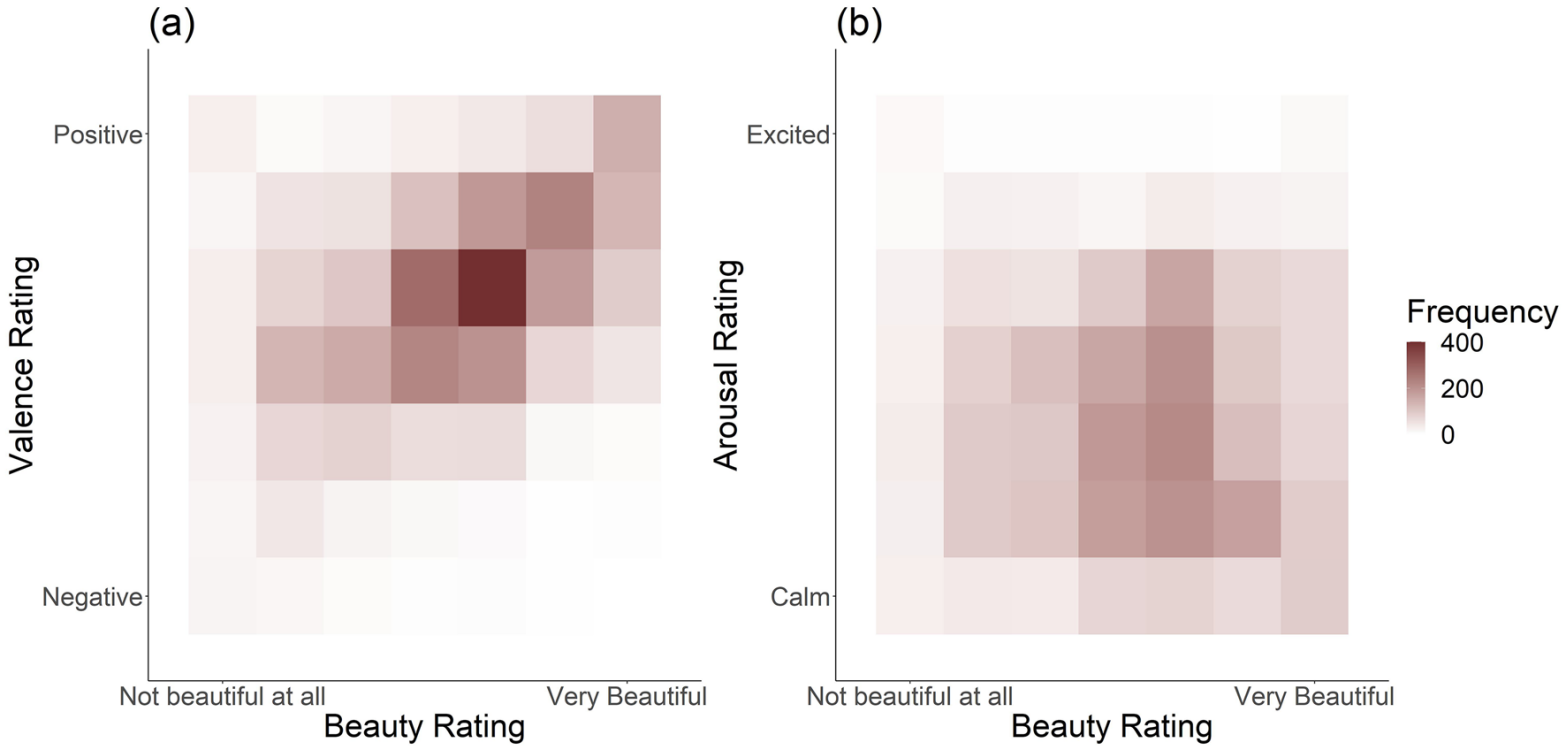
Study 3: shows frequency of occurrence of valence (**a**) or arousal ratings (**b**) in relation to different beauty ratings. Higher beauty ratings appear to more often go along with higher valence and lower arousal ratings. Overall, valence ratings tend to be medium to high; arousal ratings tend to mostly be neutral.

Lastly, the personality model shows an effect of category (b = 1.18, p < 0.001) and significant three- to five-way interactions (suppl. Table 12) (Fig. 6).

#### Influence of experiences of beauty on affective state

The results of Study 2 were replicated: More beautiful experiences receive more positive valence ratings (b = 0.30, p < 0.001, suppl. Table 13) and lower arousal ratings (b $= -0.05$, p < 0.001).

**Figure 7:**
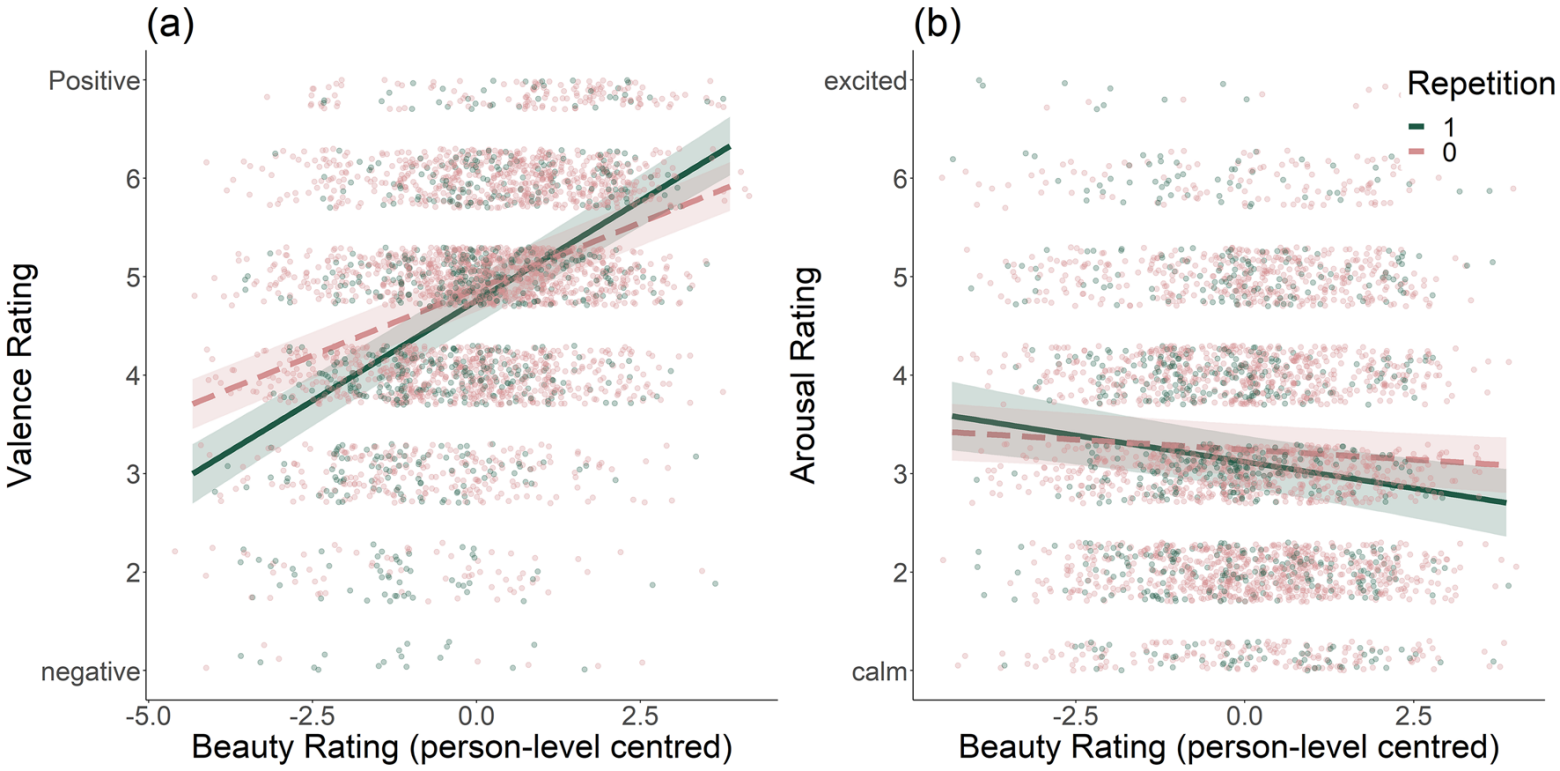
Study 3: significant interactions between beauty and repetition (1 = repeated, 0 = not repeated) of experiences on valence (**a**) and arousal (**b**). Valence: more beautiful experiences that were repeated received higher valence ratings than non-repeated experiences; less beautiful experiences received lower valence ratings than non-repeated experiences. Arousal: more beautiful experiences that were repeated received lower arousal ratings (i.e. calmer) than non-repeated experiences.

Repetition influenced both affect measures, on its own (valence: b $= -0.12$, p = 0.004); arousal: b $= -0.12$, p = 0.01), and interacting with beauty (valence: b = 0.14, p < 0.001; arousal: b $= -0.07$, p = 0.033, suppl. Table 14). We further probed these interactions using the Johnson–Neyman technique, which determines whether interactions are significant across all observed values of the predictor variables^49^. For valence, this showed that the interaction is significant, at an alpha level of .05, only if beauty ratings are below 0.27 or above 1.79 (note: these values represent the person-level centred ratings, the original scale was from 1–7). The impact of highly beautiful experiences on valence appears to be more positive if experiences are repeated; repetition of less beautiful experiences has the opposite effect. Valence is unaffected by repeated neutral (in beauty) experiences (see Fig. 7a). For arousal, the interaction is significant only for beauty ratings larger than $-0.43$, indicating that only highly beautiful repeated experiences result in lower (i.e. calmer) arousal ratings (see Fig. 7b); arousal is unaffected by the repetition of less beautiful experiences.

## General discussion

We investigated everyday aesthetic experiences in three ESM studies hypothesising that (1) such experiences are frequent, (2) natural stimuli are more likely to elicit aesthetic experiences than human-made stimuli, (3) this relation is moderated by individual differences, and (4) such experiences influence affective state. In Study 1 participants reported having had an aesthetic experience in the preceding hour in 35% of the ESM measurements; Studies 2 and 3 showed that participants rated the majority of randomly sampled experiences as highly beautiful. All three studies thus support H1, suggesting that aesthetic experiences are indeed very frequent—especially considering that in Study 3 participants received 2–3 notifications per hour.

Although stimuli we encounter in everyday life are often human-made, all three studies supported H2: natural stimuli are perceived as more beautiful and thus tend to evoke aesthetic experiences more frequently than human-made ones.

Studies 2 and 3 partially support H3, showing effects of individual differences. However, these effects are less clear and inconsistent across the two studies.

Lastly, with regard to H4, we found that experiencing beauty has a positive emotional influence with more beautiful experiences leading to more positive valence and lower (i.e., calmer) arousal ratings. Study 3 showed that repetition of these experiences may play an important moderating role. Whereas repeated (or maybe, extended) experiences with highly beautiful stimuli increase positive valence, repeated experiences with non-beautiful stimuli lead to less positive valence. For arousal, only repetition of highly beautiful stimuli results in calmer arousal states than non-repeated stimuli. However, natural fluctuations in affect were not controlled for and measures were simple to keep the ESM questionnaire brief.

Together, our findings provide the first clear support for a fundamental assumption: aesthetic experiences are frequent in daily life. Additionally, our findings suggest that natural objects are more likely to elicit aesthetic experiences, supporting the notion of beauty as a relevant factor in our evolutionary past and present. Importantly, these findings support key assumptions concerning why we developed the ability to see beauty in the first place and indicate that our sense of (natural) beauty is not merely a by-product but likely a key factor in our functioning. That said, our results cannot explain why we see beauty in human-made objects; whether this is a by-product or not will remain a topic of debate. Testing evolutionary hypotheses remains tricky; our methods can only assess one moment in time. Therefore, approaches including cross-cultural (cultural/technical evolution) or cross-species (biological evolution) research (e.g., Mühlenberg et al., 2015, 2016, 2017^50–52^) or an interdisciplinary perspective building on e.g. archaeology (e.g., symbolic artifacts^53,54^) are potential future directions to further investigate these hypotheses. Nevertheless, the results regarding H4 may provide additional insight: Though beauty may have developed to increase evolutionary success (e.g., mate or habitat selection) our results indicate that beautiful environments are rewarding in and of themselves — they makes us happy and calm us down. This can have important implications through a connection to emotional well-being (conceptualised as positive affective state), as our results indicate a positive affective influence of aesthetic experiences. These findings fit within a trend showing potential well-being effects of art and aesthetic experiences^5,28,29^ but also experiences with nature^13^. Especially in nature experiences the aesthetic aspect may play a key role. Potentially, the reason why nature increases our well-being is because we find it beautiful^55^.

In addition, as discussed in the introduction, people differ in what they find beautiful, and as shown by our results, aesthetic experiences may be influenced by individual differences. However, our results are not clear in this direction.

Concerning art- or beauty-specific individual differences (art interest, knowledge, and engagement with beauty) we found inconsistent results. Contrary to our expectations, higher art knowledge led to lower beauty ratings for both human-made and natural stimuli, in Study 2. As noted, this may represent a more critical attitude. In contrast, high art interest may bring us away from our evolutionary self: it seemed to specifically increase beauty seen in human-made objects, decreasing the difference between categories (i.e., human-made stimuli rated higher and natural lower than people with low art interest). These findings underscore the importance of focusing not on “art expertise” but on specific domains, as knowledge and interest seem to have different effects. Note however, that the effects may be caused by the sample: Novice samples (here: psychology students) show more variation in art interest than in art knowledge^40,56,57^. Thus, our sample may not include enough participants with high(er) knowledge to detect a clear or consistent difference.

Contrary to our expectations, neither Engagement with beauty nor openness to experience (which encompasses an appreciation for beauty) seemed relevant. Interestingly, this suggests that a general tendency to appreciate beauty was less important than specific interest and knowledge of one human-made category (visual art).

Future research may follow up on this, to provide further clarity on the potential cause of the individual differences, by including measures looking at either other domain-specific (e.g., music, architecture) measures or domain-general measures (e.g., aesthetic responsiveness^27^).

This connects to the also inconsistent findings regarding NR and CR relatedness. While we assumed that levels of NR or CR may strengthen our tendency to perceive beauty in either natural or human-made stimuli, respectively, our results cannot support this. As validity evidence for these scales is scarce, this may be an underlying problem. Different measures may thus lead to different results — as seen in Study 3 where the two NR measures interacted with CR in opposite directions. Nonetheless, here too, what may be more fruitful is to consider a broader question of contrasting domain-specific vs. domain-general influences.

Our results provide valuable new insight into everyday aesthetic experiences. Nonetheless, the benefit of ESM, that it accurately reflects how people actually see the world around them, also comes with limitations due to its reliance on self-report and limited experimental control. First, it cannot capture –potentially even more frequent–subconscious daily aesthetic events^39^. It would be fruitful to investigate these with other measures that do not require (conscious) self-report (e.g., mobile eye-tracking).

Second, participants were quite free in their interpretation of natural and human-made. Illustrating this problem, some participants classified photos, of e.g., gardens and parks, as human-made. Here it may be difficult to draw a clear line between the categories—they are maintained by people and contain human-made features (e.g. benches), yet their main features are usually natural (trees, grass). The opposite—largely human-made surroundings classified as natural—was also the case, occasionally (e.g., cityscapes with large portions of sky visible). With this in mind, the submitted photos may add valuable information. We currently plan to analyse these in a future study to (1) better understand what determines participants categorisations and (2) identify whether there are any commonly occurring objects that are rated highly in terms of beauty.

## Conclusion

Beauty surrounds us on a daily basis and we find it in nature, in particular. This supports beauty as a relevant factor in our evolutionary past and present. While beauty’s original usefulness might lie in increasing evolutionary success, beautiful environments appear rewarding in and of themselves—encounters with beauty have a mood-boosting impact. Further, our results indicate influences of individual differences. However, these were inconclusive and require further attention.

### Supplementary Information

Supplementary Tables.

## Data Availability

Data for Study 1 can be found here https://doi.org/10.5160/psychdata.wdre20pr28. Data for Studies 2 and 3 is available on OSF (https://osf.io/ky76r/). Participants’ photo submissions may be shared upon reasonable request only.
