# Author Correction: Experiencing beauty in everyday life

**DOI:** 10.1038/s41598-024-62206-9

**Published:** 2024-05-23

**Authors:** Anna Lena Knoll, Tristan Barrière, Rosalie Weigand, Thomas Jacobsen, Helmut Leder, Eva Specker

**Affiliations:** 1 Department of Cognition, Emotion, and Methods in Psychology, University of Vienna, Vienna, Austria; 2 Experimental Psychology Unit, Helmut Schmidt University/University of the Federal Armed Forces Hamburg, Hamburg, Germany; 3 Vienna Cognitive Science Hub, University of Vienna, Vienna, Austria

Correction to: *Scientific Reports* 10.1038/s41598-024-60091-w, published online 24 April 2024

The Acknowledgements section in the original version of this Article was incomplete.

“We thank Katinka Knoll for checking the translations of the two Nature Relatedness Scales.”

now reads:

“We thank Katinka Knoll for checking the translations of the two Nature Relatedness Scales. This research was funded in in whole or in part by the Austrian Science Fund (FWF) [doi: 10.55776/P35140].”

The original Article has been corrected.

