# Supplementary material for: Experiencing beauty in everyday life
Source: Sci Rep. 2024 Apr 24;14:9463. doi: 10.1038/s41598-024-60091-w (PMC11043425; doi:10.1038/s41598-024-60091-w)
Supplement: Supplementary file 1 — Supplementary Tables. [file 41598_2024_60091_MOESM1_ESM.pdf]

Experiencing Beauty in Everyday Life

Anna Lena Knoll[1,*], Tristan Barrière[1], Rosalie Weigand[2], Thomas Jacobsen[2], Helmut Leder[1], and Eva Specker[1]

[1]University of Vienna, Department of Cognition, Emotion, and Methods in Psychology, Vienna, Austria
[2]Helmut Schmidt University/University of the Federal Armed Forces Hamburg, Experimental Psychology Unit, Hamburg, Germany
[*]anna.lena.knoll@univie.ac.at

February 6, 2024

Supplementary tables for Study 2 and 3

		Study 2 (N = 85)	Study 3 (N = 45)
	**Response Rate** (%)		
ESM	Mean (SD)	78.6 (7.81)	80.4 (9.33)
	Median [min, max]	79.0 [55.2, 93.3]	78.0 [65.0, 100]
	**Response Duration (seconds)**		
	Mean (SD)	78.7 (29.7)	71.7 (25.0)
	Median [min, max]	72.3 [43.1, 232]	64.3 [39.9, 154]
	**Response Delay (minutes)**		
	Mean (SD)	4.07 (1.39)	2.00 (1.90)
	Median [min, max]	3.96 [1.40, 7.55]	1.78 [0.712, 13.7]
	**Event-Contingent Responses**		
	Mean (SD)	1.79 (5.09)	0.44 (1.59)
	Median [min, max]	0 [0, 38]	0 [0, 9]
	**Human-made (vs. Natural) (%)**		
	Mean (SD)	84.9 (9.2)	86.3 (9.37)
	Median [min, max]	85.2 [56.3, 100]	88.4 [61.6, 100]
	**Surroundings as a whole (vs Object within Surroundings) (%)**		
	Mean (SD)	61.6 (± 17.4)	50.8 (± 19.0)
	Median [min, max]	61.9 [18.3, 93.2]	50.7 [15.6, 91.0]
	**Photo Option Used (%)**		
	Mean (SD)	88.5 (15.4)	90.0 (14.8)
	Median [min, max]	94.4 [27.5, 100]	95.5 [17.3, 100]
	**Repeated Experiences (%)**		
	Mean (SD)		25.5 (15.7)
	Median [min, max]		23.2 [1.11, 61.3]
	**Estimated time spent in mostly human-made (vs natural) surroundings. (%)**		
post-ESM	Mean (SD)	85.0 (9.82)	86.9 (16.0)
	Median [min, max]	86.0 [51.0, 100]	91.0 [12.0, 100]
	**Mostly was in Vienna during the Study. (N (%))**	66 (77.6%)	37 (82.2%)
	**Believe they changed how they look at their surroundings. (N (%)) ***	44 (51.8%)	20 (44.4%)

* Some of the commonly reported changes:
1) Noticing nature (in urban areas) more.
2) Noticing how little time is spent in nature and wanting to change that.
3) Noticing how many of our everyday environments are human-made.
4) Being more attentive and mindful of ones surroundings generally or of beauty within ones surroundings, specifically.

Supplementary Table 1. Summary Table of 1) Response Behaviour in the ESM part of Study 2 and 3, and 2) Responses to the post-ESM questionnaire.

.

Study 2: Category Model

Predictors	*Estimates (SE)*	*CI*	*t-value*	*p*
Intercept	4.39 (0.07)	4.24 – 4.53	59.25	**<0.001**
Category	1.64 (0.05)	1.55 – 1.74	33.76	**<0.001**
Random Effects				
$\sigma 2$	2.056			
$\tau 00$ Participant	0.437			
ICC	0.175			
N Participant	85			
Observations	7163			
Marginal R2 / Conditional R2	0.123 / 0.276			

Note: Dependent Variable = Beauty Rating. Category coded as 0 = human-made and 1 = natural.

Supplementary Table 2. Study 2: Results of the multilevel model investigating the influence of category (i.e. human-made vs. natural) on experiences of beauty. Including category as a predictor significantly improved the fit of the model as compared to the null model ($\chi 2$ = 1057.2, df = 1, p < 0.001).

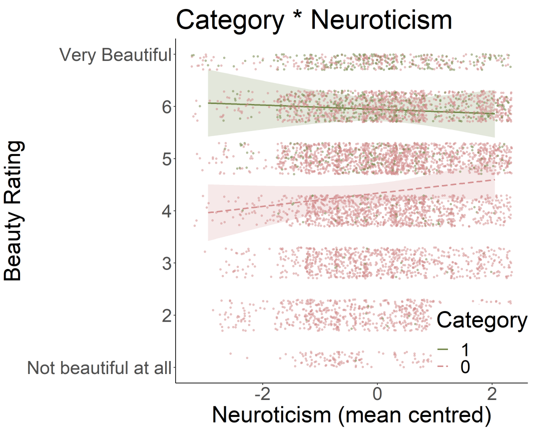

Supplementary Figure 1. Significant two-way interaction found in the 'Personality Model' in Study 2. Specifically, category (0 = human-made, 1 = natural) interacts with neuroticism in that it beauty ratings increase with higher levels of neuroticism in the human-made category and a slight decrease in the natural category.

Study 2: Aesthetics Model

Predictors	*Estimates (SE)*	*CI*	*t-value*	*p*
Intercept	4.327 (0.094)	4.142 – 4.511	46.018	**<0.001**
Category	1.625 (0.060)	1.506 – 1.743	26.885	**<0.001**
Art Knowledge (K)	-0.079 (0.037)	-0.152 – -0.006	-2.112	**0.035**
Art Interest (I)	0.015 (0.008)	-0.000 – 0.031	1.935	0.053
Engagement with Aesthetics (EBS)	0.005 (0.007)	-0.009 – 0.019	0.648	0.517
Category*K	0.051 (0.022)	0.007 – 0.094	2.268	**0.023**
Category*I	-0.025 (0.005)	-0.035 – -0.016	-5.091	**<0.001**
K*I	0.001 (0.002)	-0.004 – 0.005	0.304	0.761
Category*EBS	0.006 (0.005)	-0.003 – 0.016	1.368	0.171
K*EBS	-0.002 (0.003)	-0.008 – 0.003	-0.819	0.413
I*EBS	0.001 (0.001)	-0.0004 – 0.0016	1.125	0.261
Category*K*I	0.003 (0.001)	0.0004 – 0.0059	2.261	**0.024**
Category*K*EBS	0.004 (0.002)	0.0004 – 0.0079	2.175	**0.030**
Category*I*EBS	-0.0005 (0.0004)	-0.0012 – 0.0002	-1.326	0.185
K*I*EBS	0.0002 (0.0001)	-0.0001 – 0.0005	1.298	0.194
Category*K*I*EBS	-0.0003 (0.0001)	-0.0005 – -0.0001	-3.390	**0.001**
Random Effects				
$\sigma 2$	2.042			
$\tau 00$ (Participant)	0.440			
ICC	0.177			
N	85			
Observations	7163			
Marginal R2 / Conditional R2	0.145 / 0.296			

Supplementary Table 3. Study 2: Aesthetics Model. The inclusion of Art Interest, Art Knowledge, and Engagement with Beauty significantly improved the model fit compared to the 'category model' ($\chi 2$ = 63.21, df = 14, p < 0.001).

Study 2: Nature and City Relatedness Model

Predictors	*Estimates*	*CI*	*t-value*	*p*
Intercept	4.318 (0.103)	4.115 – 4.521	41.770	**<0.001**
Category	1.607 (0.067)	1.475 – 1.739	23.848	**<0.001**
Nature Relatedness Scale (NR)	0.226 (0.278)	-0.318 – 0.771	0.815	0.415
Nature Connection Index (NCI)	-0.001 (0.005)	-0.012 – 0.009	-0.237	0.813
City Relatedness Scale (CR)	0.145 (0.195)	-0.237 – 0.527	0.743	0.457
Category*NR	0.159 (0.189)	-0.212 – 0.529	0.841	0.401
Category*NCI	-0.001 (0.004)	-0.007 – 0.006	-0.169	0.866
NR*NCI	0.008 (0.007)	-0.007 – 0.022	1.050	0.294
Category*CR	-0.085 (0.118)	-0.316 – 0.147	-0.716	0.474
NR*CR	-0.194 (0.529)	-1.232 – 0.843	-0.367	0.714
NCI*CR	0.006 (0.009)	-0.011 – 0.023	0.669	0.504
Category*NR*NCI	0.002 (0.005)	-0.009 – 0.013	0.385	0.700
Category*NR*CR	0.336 (0.315)	-0.281 – 0.953	1.067	0.286
Category*NCI*CR	-0.003 (0.006)	-0.014 – 0.008	-0.577	0.564
NR*NCI*CR	0.002 (0.018)	-0.032 – 0.037	0.140	0.889
Category*NR*NCI*CR	-0.003 (0.011)	-0.025 – 0.020	-0.223	0.823
Random Effects				
$\sigma 2$	2.057			
$\tau 00$ (Participant)	0.449			
ICC	0.179			
N	85			
Observations	7163			
Marginal R2 / Conditional R2	0.130 / 0.286			

Supplementary Table 4. Study 2: Nature and City Relatedness Model. In contrast to the aesthetics model, the nature and city relatedness model did not significantly improve the model fit compared to the 'category model' ($\chi 2$ = 8.5, df = 14, p = 0.86).

Study 2: Personality Model

Predictors	*Estimates*	*CI*	*t-value*	*p-value*
Intercept	4.339 (0.097)	4.149 – 4.529	44.777	**<0.001**
Category	1.600 (0.070)	1.462 – 1.738	22.710	**<0.001**
Openness to Experience (O)	-0.006 (0.098)	-0.198 – 0.185	-0.062	0.951
Agreeableness (A)	-0.095 (0.142)	-0.373 – 0.183	-0.668	0.504
Conscientiousness (C)	-0.019 (0.136)	-0.285 – 0.248	-0.138	0.890
Neuroticism (N)	0.133 (0.088)	-0.040 – 0.307	1.511	0.131
Extraversion (E)	0.120 (0.088)	-0.053 – 0.292	1.361	0.173
Category*O	-0.036 (0.068)	-0.170 – 0.097	-0.531	0.596
Category*A	0.123 (0.091)	-0.056 – 0.302	1.343	0.179
O*A	-0.078 (0.139)	-0.349 – 0.194	-0.560	0.576
Category*C	0.071 (0.098)	-0.121 – 0.263	0.726	0.468
O*C	0.039 (0.120)	-0.196 – 0.274	0.328	0.743
A*C	0.205 (0.136)	-0.062 – 0.472	1.506	0.132
Category*N	-0.167 (0.065)	-0.295 – -0.040	-2.571	**0.010**
O*N	-0.085 (0.117)	-0.313 – 0.144	-0.725	0.468
A*N	0.002 (0.146)	-0.284 – 0.289	0.017	0.987
C*N	-0.100 (0.159)	-0.411 – 0.211	-0.632	0.528
Category*E	-0.070 (0.059)	-0.186 – 0.047	-1.172	0.241
O*E	-0.104 (0.079)	-0.259 – 0.052	-1.304	0.192
A*E	0.013 (0.121)	-0.225 – 0.251	0.109	0.913
C*E	0.148 (0.127)	-0.100 – 0.396	1.170	0.242
N*E	0.097 (0.077)	-0.054 – 0.247	1.255	0.210
Category*O*A	0.035 (0.090)	-0.143 – 0.212	0.382	0.703
Category*O*C	-0.024 (0.099)	-0.217 – 0.170	-0.239	0.811
Category*A*C	-0.250 (0.095)	-0.436 – -0.065	-2.642	**0.008**
O*A*C	0.088 (0.134)	-0.175 – 0.350	0.656	0.512
Category*O*N	0.182 (0.085)	0.014 – 0.349	2.127	**0.033**
Category*A*N	-0.143 (0.115)	-0.369 – 0.083	-1.238	0.216
O*A*N	-0.123 (0.206)	-0.527 – 0.282	-0.594	0.553
Category*C*N	0.028 (0.110)	-0.189 – 0.245	0.254	0.799
O*C*N	0.121 (0.135)	-0.143 – 0.385	0.900	0.368
A*C*N	-0.078 (0.170)	-0.411 – 0.255	-0.459	0.646
Category*O*E	0.133 (0.052)	0.031 – 0.236	2.555	**0.011**
Category*A*E	-0.006 (0.085)	-0.172 – 0.160	-0.070	0.944
O*A*E	-0.112 (0.135)	-0.377 – 0.152	-0.832	0.405
Category*C*E	0.010 (0.091)	-0.169 – 0.190	0.115	0.909
O*C*E	0.037 (0.115)	-0.189 – 0.264	0.325	0.745
A*C*E	0.134 (0.128)	-0.117 – 0.385	1.048	0.294
Category*N*E	-0.043 (0.051)	-0.142 – 0.057	-0.836	0.403
O*N*E	0.071 (0.083)	-0.092 – 0.234	0.853	0.394
A*N*E	-0.154 (0.120)	-0.389 – 0.081	-1.287	0.198
C*N*E	-0.103 (0.151)	-0.398 – 0.193	-0.681	0.496

Predictors	*Estimates*	*CI*	*t-value*	*p-value*
Category*O*A*E	-0.262 (0.111)	-0.479 – -0.045	-2.370	**0.018**
Category*O*A*N	-0.222 (0.181)	-0.578 – 0.134	-1.223	0.221
Category*O*C*N	0.017 (0.093)	-0.165 – 0.200	0.186	0.853
Category*A*C*N	0.090 (0.132)	-0.168 – 0.348	0.682	0.495
O*A*C*N	0.072 (0.221)	-0.362 – 0.505	0.324	0.746
Category*O*A*E	-0.134 (0.097)	-0.325 – 0.057	-1.378	0.168
Category*O*C*E	0.059 (0.085)	-0.107 – 0.225	0.699	0.485
Category*A*C*E	-0.0002 (0.097)	-0.191 – 0.191	-0.003	0.998
O*A*C*E	0.115 (0.142)	-0.163 – 0.393	0.811	0.418
Category*O*N*E	0.054 (0.059)	-0.062 – 0.171	0.910	0.363
Category*A*N*E	0.185 (0.087)	0.016 – 0.355	2.141	**0.032**
O*A*N*E	0.252 (0.170)	-0.082 – 0.586	1.479	0.139
Category*C*N*E	0.014 (0.102)	-0.187 – 0.214	0.133	0.894
O*C*N*E	-0.055 (0.128)	-0.305 – 0.195	-0.432	0.665
A*C*N*E	0.054 (0.131)	-0.202 – 0.310	0.414	0.679
Category*O*A*C*N	-0.008 (0.200)	-0.400 – 0.384	-0.040	0.968
Category*O*A*C*E	-0.281 (0.130)	-0.537 – -0.026	-2.159	**0.031**
Category*O*A*N*E	0.113 (0.145)	-0.171 – 0.397	0.782	0.434
Category*O*C*N*E	-0.032 (0.084)	-0.198 – 0.133	-0.384	0.701
Category*A*C*N*E	-0.325 (0.120)	-0.560 – -0.091	-2.723	**0.006**
O*A*C*N*E	-0.090 (0.205)	-0.493 – 0.312	-0.440	0.660
Category*O*A*C*N*E	-0.460 (0.225)	-0.901 – -0.020	-2.047	**0.041**
Random Effects				
σ2	2.056			
τ00 (Participant)	0.470			
ICC	0.186			
N	84			
Observations	7076			
Marginal R2 / Conditional R2	0.168 / 0.323			

Supplementary Table 5. Study 2: Personality Model. The inclusion of the personality facets as predictors significantly improved the model fit compare to the 'category model' ($\chi 2$ = 101.28, df = 62, p = 0.001).

	**Study 2 (N = 85)**	**Study 3 (N = 45)**
Scale	Mean (SD)	Mean (SD)
**VAIAK**		
Art Interest	40.3 (± 13.1)	39.8 (± 12.5)
Art Knowledge	6.18 (± 3.30)	6.18 (± 3.48)
**NCI**	60.6 (± 25.2)	58.2 (± 21.7)
**NRS**	3.71 (± 0.48)	3.68 (± 0.49)
perspective	4.19 (± 0.5)	4.10 (± 0.47)
self	3.60 (± 0.61)	3.56 (± 0.63)
experience	3.30 (± 0.7)	3.34 (± 0.81)
**CRS**	3.03 (± 0.58)	3.03 (± 0.52)
self	2.78 (± 0.67)	2.81 (± 0.61)
experience	3.36 (± 0.63)	3.33 (± 0.59)
**EBS**	66.7 (± 13.4)	66.3 (± 13.8)
artistic	20.5 (± 4.67)	16.1 (± 5.19)
natural	15.6 (± 5.28)	20.4 (± 4.91)
moral	30.7 (± 6.35)	29.7 (± 7.09)
**BFI**		
Openness to Experience	3.82 (± 1.16)	3.83 (± 0.97)
Conscientiousness	3.68 (± 0.92)	3.33 (± 1.04)
Agreeableness	3.46 (± 0.96)	3.72 (± 0.9)
Extraversion	3.25 (± 1.29)	3.24 (± 1.31)
Neuroticism	3.51 (± 1.16)	3.63 (± 1.29)

Note: for Study 2 Openness to Experience N = 84, as one person seems to have skipped an item in the BFI.

Supplementary Table 6. Individual Difference Measures. Mean scores and standard deviations for all individual difference measures and their subscales are shown for both Study 2 (left) and 3 (right).

	Study 2: Valence Model				Study 2: Arousal Model			
Predictors	*Estimates (SE)*	*CI*	*t-value*	*p*	*Estimates (SE)*	*CI*	*t-value*	*p*
Intercept	5.02 (0.08)	4.86 – 5.17	64.56	**<0.001**	3.08 (0.09)	2.91 – 3.25	34.98	**<0.001**
Beauty	0.29 (0.01)	0.27 – 0.31	36.63	**<0.001**	-0.09 (0.01)	-0.11 – -0.07	-8.65	**<0.001**
Random Effects								
σ2	1.058				1.689			
τ00 Participant	0.455				0.624			
τ00 Day	0.004				0.001			
ICC	0.303				0.270			
N Participant	85				85			
N Day	7				7			
Observations	7162				7163			
Marginal R2 / Conditional R2	0.116 / 0.383				0.008 / 0.276			

Supplementary Table 7. Study 2: Results of the two multilevel models investigating the influence of experiences of beauty on the two mood measures (i.e. valence and arousal). Compared to their respective null models (i.e. only including participant and day as random intercepts), inclusion of beauty as a fixed effect term improves model fit of the valence ($\chi 2$ = 1229.04, df = 1, p < 0.001) and arousal models ($\chi 2$ = 74.39, df = 1, p < 0.001)

	Study 3: Category Model			
Predictors	*Estimates (SE)*	*CI*	*t-value*	*p*
Intercept	4.21 (0.09)	4.03 – 4.39	44.76	**<0.001**
Category	1.64 (0.07)	1.50 – 1.78	22.90	**<0.001**
Random Effects				
σ2	2.033			
τ00 Participant	0.368			
ICC	0.153			
N Participant	45			
Observations	3634			
Marginal R2 / Conditional R2	0.116 / 0.251			

Supplementary Table 8. Study 3: Results of the multilevel model investigating the influence of category (i.e. human-made vs. natural) on experiences of beauty. Including category as a predictor significantly improved the fit of the model as compared to the null model ($\chi 2$ = 490.22, df = 1, p < 0.001).

	Study 3: Category and Repetition Model			
Predictors	*Estimates*	*CI*	*t-value*	*p*
Intercept	4.26 (0.10)	4.07 – 4.44	44.57	**<0.001**
Category	1.57 (0.08)	1.42 – 1.72	20.25	**<0.001**
Repetition	-0.17 (0.06)	-0.28 – -0.05	-2.73	**0.006**
Category*Repetition	0.34 (0.20)	-0.05 – 0.72	1.72	0.086
Random Effects				
σ2	2.030			
τ00 Participant	0.368			
ICC	0.154			
N Participant	45			
Observations	3634			
Marginal R2 / Conditional R2	0.118 / 0.253			

Supplementary Table 9. Study 3: Results of the multilevel model investigating the influence of category (i.e. human-made vs. natural) and repetition on experiences of beauty. Including repetition additionally improved the model fit compared to the model including only category ($\chi 2$ = 8.36, df = 2, p = 0.015)

Study 3: Aesthetics Model

Predictors	*Estimates (SE)*	*CI*	*t-value*	*p*
Intercept	4.3599 (0.1091)	4.1461 – 4.5738	39.9693	**<0.001**
Category	1.6281 (0.0852)	1.4611 – 1.7951	19.1115	**<0.001**
Art Knowledge (K)	-0.0118 (0.0380)	-0.0864 – 0.0628	-0.3104	0.756
Art Interest (I)	0.0033 (0.0091)	-0.0146 – 0.0212	0.3586	0.720
Engagement with Aesthetics (EBS)	0.0066 (0.0076)	-0.0084 – 0.0215	0.8615	0.389
Category*K	-0.0258 (0.0297)	-0.0841 – 0.0325	-0.8675	0.386
Category*I	-0.0017 (0.0075)	-0.0165 – 0.0131	-0.2298	0.818
K*I	-0.0086 (0.0030)	-0.0145 – -0.0028	-2.8876	**0.004**
Category*EBS	-0.0056 (0.0064)	-0.0181 – 0.0070	-0.8674	0.386
K*EBS	0.0006 (0.0033)	-0.0058 – 0.0070	0.1835	0.854
I*EBS	0.0005 (0.0006)	-0.0007 – 0.0016	0.8105	0.418
Category*K*I	0.0025 (0.0024)	-0.0022 – 0.0073	1.0372	0.300
Category*K*EBS	-0.0006 (0.0025)	-0.0055 – 0.0042	-0.2545	0.799
Category*I*EBS	-0.0004 (0.0005)	-0.0013 – 0.0005	-0.8676	0.386
K*I*EBS	0.0002 (0.0002)	-0.0003 – 0.0006	0.8105	0.418
Category*K*I*EBS	-0.0000 (0.0002)	-0.0004 – 0.0004	-0.0076	0.994
Random Effects				
$\sigma 2$	2.034			
$\tau 00$ (Participant)	0.338			
ICC	0.143			
N	45			
Observations	3634			
Marginal R2 / Conditional R2	0.153 / 0.273			

Supplementary Table 10. Study 3: Aesthetics Model. Model fit was not improved over that of the category only model ($\chi 2$ = 16.90, df = 14, p = 0.261). Removing EBS from the model, as here it does not even appear relevant in 3- or 4-way interactions as it did in study 2, did not significantly improve model fit either.

Study 3: Nature and City Relatedness Model

Predictors	*Estimates*	*CI*	*t-value*	*p*
Intercept	4.180 (0.109)	3.967 – 4.394	38.441	**<0.001**
Category	1.569 (0.091)	1.391 – 1.746	17.319	**<0.001**
Nature Relatedness Scale (NR)	0.019 (0.281)	-0.532 – 0.570	0.068	0.946
Nature Connection Index (NCI)	-0.001 (0.006)	-0.014 – 0.011	-0.230	0.818
City Relatedness Scale (CR)	0.131 (0.223)	-0.306 – 0.567	0.586	0.558
Category*NR	-0.083 (0.252)	-0.577 – 0.412	-0.329	0.743
Category*NCI	0.006 (0.006)	-0.005 – 0.018	1.104	0.270
NR*NCI	0.004 (0.009)	-0.013 – 0.021	0.419	0.675
Category*CR	-0.027 (0.172)	-0.365 – 0.310	-0.158	0.875
NR*CR	-1.370 (0.632)	-2.610 – -0.130	-2.167	**0.030**
NCI*CR	0.026 (0.012)	0.003 – 0.049	2.194	**0.028**
Category*NR*NCI	0.011 (0.008)	-0.004 – 0.026	1.441	0.150
Category*NR*CR	0.117 (0.509)	-0.881 – 1.114	0.229	0.819
Category*NCI*CR	-0.016 (0.010)	-0.035 – 0.003	-1.653	0.098
NR*NCI*CR	0.045 (0.020)	0.006 – 0.084	2.273	**0.023**
Category*NR*NCI*CR	-0.052 (0.019)	-0.089 – -0.015	-2.784	**0.005**
Random Effects				
$\sigma 2$	2.022			
$\tau 00$ (Participant)	0.331			
ICC	0.141			
N	45			
Observations	3634			
Marginal R2 / Conditional R2	0.148 / 0.268			

Supplementary Table 11. Study 3: Nature and City Relatedness Model. Model fit significantly improved compared to the category only model ($\chi 2$ = 38.87, df = 14 p < 0.001).

Study 3: Personality Model

Predictors	*Estimates*	*CI*	*t-value*	*p-value*	*Predictors*	*Estimates*	*CI*	*t-value*	*p-value*
Intercept	4.391 (0.215)	3.970 – 4.812	20.463	**<0.001**	Category*O*A*E	-0.383 (0.526)	-1.415 – 0.648	-0.728	0.467
Category	1.178 (0.144)	0.896 – 1.461	8.176	**<0.001**	Category*O*A*N	-0.176 (0.203)	-0.575 – 0.223	-0.863	0.388
Openness to Experience (O)	0.038 (0.307)	-0.563 – 0.639	0.125	0.901	Category*O*C*N	0.518 (0.222)	0.084 – 0.953	2.338	**0.019**
Agreeableness (A)	0.157 (0.280)	-0.392 – 0.706	0.561	0.575	Category*A*C*N	0.175 (0.190)	-0.198 – 0.547	0.918	0.358
Conscientiousness (C)	0.064 (0.246)	-0.418 – 0.546	0.260	0.795	O*A*C*N	-0.357 (0.639)	-1.610 – 0.895	-0.559	0.576
Neuroticism (N)	0.074 (0.152)	-0.223 – 0.371	0.487	0.626	Category*O*A*E	-0.065 (0.275)	-0.605 – 0.475	-0.237	0.813
Extraversion (E)	-0.191 (0.169)	-0.522 – 0.140	-1.132	0.258	Category*O*C*E	0.101 (0.279)	-0.446 – 0.648	0.361	0.718
Category*O	0.174 (0.207)	-0.232 – 0.580	0.842	0.400	Category*A*C*E	0.326 (0.348)	-0.356 – 1.008	0.938	0.348
Category*A	0.169 (0.198)	-0.219 – 0.556	0.855	0.393	O*A*C*E	0.524 (0.642)	-0.735 – 1.782	0.816	0.415
O*A	0.506 (0.439)	-0.355 – 1.367	1.152	0.249	Category*O*N*E	0.055 (0.121)	-0.183 – 0.292	0.452	0.651
Category*C	-0.181 (0.179)	-0.531 – 0.169	-1.013	0.311	Category*A*N*E	-0.054 (0.259)	-0.563 – 0.454	-0.209	0.834
O*C	0.544 (0.398)	-0.237 – 1.324	1.366	0.172	O*A*N*E	-0.182 (0.239)	-0.650 – 0.286	-0.764	0.445
A*C	-0.192 (0.383)	-0.944 – 0.560	-0.501	0.616	Category*C*N*E	-0.228 (0.192)	-0.605 – 0.150	-1.184	0.237
Category*N	0.093 (0.118)	-0.139 – 0.325	0.787	0.431	O*C*N*E	0.025 (0.250)	-0.466 – 0.515	0.100	0.921
O*N	-0.195 (0.240)	-0.666 – 0.275	-0.814	0.416	A*C*N*E	-0.041 (0.313)	-0.655 – 0.573	-0.130	0.896
A*N	-0.080 (0.223)	-0.516 – 0.356	-0.359	0.719	Category*O*A*C*N	0.670 (0.428)	-0.169 – 1.510	1.565	0.118
C*N	-0.303 (0.289)	-0.870 – 0.264	-1.046	0.295	Category*O*A*C*E	-0.891 (0.463)	-1.799 – 0.017	-1.924	0.054
Category*E	0.159 (0.098)	-0.032 – 0.351	1.631	0.103	Category*O*A*N*E	0.348 (0.201)	-0.046 – 0.742	1.732	0.083
O*E	-0.113 (0.230)	-0.564 – 0.338	-0.492	0.623	Category*O*C*N*E	0.356 (0.175)	0.014 – 0.699	2.038	**0.042**
A*E	-0.193 (0.276)	-0.734 – 0.347	-0.701	0.483	Category*A*C*N*E	0.184 (0.223)	-0.252 – 0.621	0.828	0.408
C*E	0.013 (0.199)	-0.376 – 0.403	0.066	0.947	O*A*C*N*E	-0.063 (0.327)	-0.705 – 0.579	-0.191	0.848
N*E	-0.136 (0.164)	-0.458 – 0.186	-0.828	0.408	Category*O*A*C*N*E	0.112 (0.223)	-0.325 – 0.549	0.504	0.615
Category*O*A	-0.309 (0.320)	-0.937 – 0.319	-0.964	0.335	*Random Effects*				
Category*O*C	-0.434 (0.294)	-1.010 – 0.141	-1.480	0.139	σ2	2.023			
Category*A*C	0.017 (0.275)	-0.522 – 0.556	0.061	0.952	τ00 (Participant)	0.540			
O*A*C	0.484 (0.797)	-1.078 – 2.045	0.607	0.544	ICC	0.211			
Category*O*N	0.408 (0.162)	0.090 – 0.727	2.515	**0.012**	N	45			
Category*A*N	0.162 (0.159)	-0.150 – 0.473	1.018	0.309	Observations	3634			
O*A*N	0.048 (0.305)	-0.549 – 0.646	0.159	0.874	Marginal R2 / Conditional R2	0.185 / 0.357			
Category*C*N	0.041 (0.209)	-0.369 – 0.451	0.196	0.844					
O*C*N	-0.560 (0.327)	-1.201 – 0.082	-1.711	0.087					
A*C*N	-0.269 (0.267)	-0.792 – 0.255	-1.005	0.315					
Category*O*E	0.102 (0.172)	-0.236 – 0.441	0.594	0.552					
Category*A*E	-0.173 (0.208)	-0.581 – 0.235	-0.830	0.406					
O*A*E	-0.092 (0.360)	-0.797 – 0.614	-0.254	0.799					
Category*C*E	-0.026 (0.124)	-0.270 – 0.218	-0.208	0.835					
O*C*E	-0.157 (0.334)	-0.811 – 0.497	-0.471	0.637					
A*C*E	-0.165 (0.501)	-1.147 – 0.817	-0.330	0.742					
Category*N*E	-0.030 (0.098)	-0.223 – 0.163	-0.308	0.758					
O*N*E	-0.096 (0.164)	-0.418 – 0.226	-0.583	0.560					
A*N*E	-0.142 (0.355)	-0.839 – 0.555	-0.400	0.689					
C*N*E	0.107 (0.271)	-0.425 – 0.639	0.394	0.694					

Supplementary Table 12. Study 3: Personality Model. Model fit significantly improved compared to the category only model ($\chi 2$ = 89.07, df = 62, p = 0.014).

	**Study 3: Valence Model**				**Study 3: Arousal Model**			
Predictors	*Estimates (SE)*	*CI*	*t-value*	*p*	*Estimates (SE)*	*CI*	*t-value*	*p*
Intercept	4.84 (0.12)	4.61 – 5.07	41.43	**<0.001**	3.22 (0.13)	2.96 – 3.47	24.63	**<0.001**
Beauty	0.30 (0.01)	0.28 – 0.32	27.85	**<0.001**	-0.05 (0.01)	-0.08 – -0.03	-4.19	**<0.001**
Random Effects								
σ2	0.989				1.381			
τ00 Participant	0.485				0.688			
τ00 Day	0.005				0.003			
ICC	0.331				0.333			
N Participant	45				45			
N Day	2				2			
Observations	3634				3634			
Marginal R2 / Conditional R2	0.125 / 0.415				0.003 / 0.335			

Supplementary Table 13. Study 3: Results of the two multilevel models investigating the influence of experiences of beauty on the two mood measures (i.e. valence and arousal). Compared to their respective null models (i.e. only including participant and day as random intercepts), inclusion of beauty as a fixed effect term improves model fit of the valence ($\chi 2$ = 702.56, df = 1, $p < 0.001$) and arousal models ($\chi 2$ = 17.49, df = 1, $p < 0.001$).

	Study 3: Valence and Repetition Model				Study 3: Arousal and Repetition Model			
Predictors	*Estimates (SE)*	*CI*	*t-value*	*p*	*Estimates (SE)*	*CI*	*t-value*	*p*
Intercept	4.88 (0.12)	4.65 – 5.10	41.84	**<0.001**	3.24 (0.13)	2.99 – 3.50	24.60	**<0.001**
Beauty	0.27 (0.01)	0.25 – 0.29	21.75	**<0.001**	-0.04 (0.01)	-0.07 – -0.01	-2.78	**0.005**
Repetition	-0.12 (0.04)	-0.20 – -0.04	-2.92	**0.004**	-0.12 (0.05)	-0.22 – -0.03	-2.59	**0.010**
Beauty*Repetition	0.14 (0.03)	0.08 – 0.19	5.19	**<0.001**	-0.07 (0.03)	-0.13 – -0.01	-2.13	**0.033**
Random Effects								
$\sigma 2$	0.980				1.378			
$\tau 00$ Participant	0.471				0.693			
$\tau 00$ Day	0.005				0.003			
ICC	0.327				0.336			
N Participant	45				45			
N Day	2				2			
Observations	3634				3634			
Marginal R2 / Conditional R2	0.132 / 0.416				0.005 / 0.339			

Supplementary Table 14. Study 3: Results of the two multilevel models investigating the influence of experiences of beauty and repetition on the two mood measures (i.e. valence and arousal). Adding repetition into the models further improves the model fit (valence: $\chi 2 = 37.71$, df = 2, $p < 0.001$; arousal: $\chi 2 = 10.52$, df = 2, $p = 0.005$).
